# Comparison of false positive and false negative rates of two indices of individual reliable change: Jacobson-Truax and Hageman-Arrindell methods

**DOI:** 10.3389/fpsyg.2023.1132128

**Published:** 2023-07-13

**Authors:** Rodrigo Ferrer-Urbina, Antonio Pardo, Willem A. Arrindell, Giannina Puddu-Gallardo

**Affiliations:** ^1^Universidad de Tarapacá, Av. General Velásquez, Arica, Chile; ^2^Universidad Autónoma de Madrid, Ciudad Universitaria de Cantoblanco, Madrid, Spain; ^3^University of Social Sciences and Humanities, Vietnam National University, Ho Chi Minh City, Vietnam

**Keywords:** individual reliable change, assessment of change, Jacobson-Truax method, Hageman-Arrindell approach, false negatives, false positives

## Abstract

**Background:**

Quantification of change is crucial for correctly estimating the effect of a treatment and for distinguishing random or non-systematic changes from substantive changes. The objective of the present study was to learn about the performance of two distribution-based methods [the Jacobson-Truax Reliable Change Index (RCI) and the Hageman-Arrindell (HA) approach] that were designed for evaluating individual reliable change.

**Methods:**

A pre-post design was simulated with the purpose to evaluate the false positive and false negative rates of RCI and HA methods. In this design, a first measurement is obtained before treatment and a second measurement is obtained after treatment, in the same group of subjects.

**Results:**

In relation to the rate of false positives, only the HA statistic provided acceptable results. Regarding the rate of false negatives, both statistics offered similar results, and both could claim to offer acceptable rates when Ferguson’s stringent criteria were used to define effect sizes as opposed to when the conventional criteria advanced by Cohen were employed.

**Conclusion:**

Since the HA statistic appeared to be a better option than the RCI statistic, we have developed and presented an Excel macro so that the greater complexity of calculating HA would not represent an obstacle for the non-expert user.

## Introduction

In the field of applied research, having objective and reliable measures to assess the change experienced after an intervention is crucial, for example in a clinical context, the interpretation of the results of a treatment will influence clinical decision-making, including the safety and efficacy of the patient (Page, 2014). In recent decades, there has been an increase in pre-post study designs that include measures to assess the efficacy of an intervention or treatment, in an effort to redirect practice in a more oriented to “Evidence-Based Practice” (Page, 2014). The pre-post design studies are especially useful in the clinical context since they allow to measure the variations in a variable of interest (e.g., depression and/or anxiety symptoms, consumption patterns of any substance, etc.) before and after an intervention and therefore evaluate the success of the strategy used, like to define the clinically meaningful change in the GAD-7 scale (Bischoff et al., 2020); compare different treatment approaches as multi-family groups (MFG) (Vardanian et al., 2020); or assess clinical change in mental health with psychiatric patients (Shalaby et al., 2022). Although the quantification of this change is essential to correctly estimate the effect of a treatment, this itself is not enough since it must also be able to distinguish random or non-systematic changes from substantive changes.

In this context, a clinician or researcher could draw any of four conclusions: correctly conclude that change has taken place (true positive); correctly conclude that no change has taken place (true negative); erroneously conclude that significant change has taken place (the result is positive), when in reality such a change has not taken place (false positive); or erroneously conclude that no significant change has taken place (the result is negative), when in reality meaningful change has taken place (false negative).

Among the available strategies for assessing change, *distribution-based methods* are the most used (see 1). These are a set of techniques designed for identifying clinically meaningful change, based on the statistical properties of magnitude estimates of change and data variability, this mean that, can be estimated based on the distribution of observed scores in a relevant sample (Revicki et al., 2008).

These methods have been designed in the context of assessing clinically meaningful change to identify reliable change, i.e., minimum variations that should occur in the patients’ answers to be able to conclude that significant change has been made (McGlinchey et al., 2002; Crosby et al., 2003; Gatchel and Mayer, 2010; Turner et al., 2010). To accomplish this purpose, distribution-based methods must be able to identify those substantive changes (true positive) other than randomness from randomly attributable changes (true negative).

For these reasons, some studies were conducted to compare the accuracy of the performance of the different methods by identifying misclassifications in simulated scenarios, specifically the quantity of changes detected when the variations were only random (false positive) (Pardo and Ferrer, 2013) and the amount of undetected changes when the variations were systematic (false negative) (Ferrer and Pardo, 2019).

More than three decades have elapsed since Jacobson, Follette and Revenstorf (Jacobson et al., 1984) proposed the *reliable change index* (RCI) for assessing *individual change* as an alternative to the assessment of *group change* offered by the classical null hypothesis significance tests and measures of effect size. Along these years, the RCI has undergone some corrections by his own promoters (Jacobson and Truax, 1991; Jacobson et al., 1999) and many other researchers have proposed alternatives procedures for trying to improve accuracy and effectiveness in identifying significant or reliable changes (see, for example, Nunnally and Kotsch, 1983; Christensen and Mendoza, 1986; Hsu, 1989, 1995, 1996; Speer, 1992; Crawford and Howell, 1998; Hageman and Arrindell, 1999; Maassen, 2004; Wyrwich, 2004; Crawford and Garthwaite, 2006; Botella et al., 2018). It is important to emphasize that to estimate these measures of individual change, we require a distribution of observed data as a reference, which could be obtained from previous studies or field-related reference studies.

Despite the alternative proposals, the RCI statistic has become the most widely used index for assessing individual change in pre-post designs in clinical settings (according to “Web of Science,” the Jacobson and Truax paper (Jacobson and Truax, 1991) has received 6,843 citations until December 2022). However, the fact that a method is widely used does not mean that it is free of problems. In a study designed to assess the performance of different indices of individual change, Ferrer and Pardo (2019) have shown that false positive rates obtained with RCI are unacceptably high: depending on the context, these rates oscillate between 0 and 39.7% (between 5.0 and 34.3% when working with normal distributions), when in fact the expected values due to the cutoff points established should be around 5%.

### *RCI* versus *HA*

Several researchers have proposed similar methods to RCI in an attempt to improve their performance (for a review, see Crosby et al., 2003; Ferrer and Pardo, 2019). Many of these methods have been compared with each other to determine whether or not they made equivalent classifications; and results of these studies have shown some consistency (Estrada et al., 2019, 2020).

McGlinchey et al. (2002) compared five distribution-based methods: the reliable change index (RCI) (Jacobson et al., 1984); the Edwards-Nunnally method (EN) (Speer, 1992); the Gulliksen-Lord-Novick method (GLN) (Hsu, 1989; Maassen, 2004); a method based on the hierarchical linear modeling (HLM) (Speer, 1992); and the Hageman-Arrindell method (HA) (Hageman and Arrindell, 1999). McGlinchey et al. (2002) concluded that all methods offer similar results, with the exception of the HA method, which tends to be more conservative, this means that it tends to identify fewer changes than the other methods: “…there will need to be relatively greater change with the HA method for an individual to be considered reliably improved” (p. 543).

In a similar way, Ronk et al. (2012) found that the HA method yielded different results from the rest of the methods studied (RCI, GLN, EN, and NK) (22), which offered similar performances to each other. Bauer et al. (2004), after comparing five distribution-based methods (RCI, GLN, EN, HLM and HA), conclude that the HA method is “the most conservative.” Despite that Ronk et al. (2016), in a comparative study between RCI and HA, conclude that there is no discernible advantage in the use of one method over the other, the results reported in this study (see Table 3, p. 5) shown that the percentage of patients classified as “recovered” were systematically lower with the HA method than with the RCI method.

The results obtained on these empirical studies are in close agreement with those obtained from simulation studies. Atkins et al. (2005) have found that “the HA method is the most conservative” (p. 986) of the four compared (RCI, GLN, EN, HA), i.e., it is the method that classifies less cases as recovered. Indeed, Pardo and Ferrer (2013) have shown that, although both RCI and HA offer unacceptably high false positive rates, the HA method offers a rate systematically lower than the one obtained with RCI.

In this context, one may wonder what makes HA work differently from RCI and other distribution-based methods. We believe that the answer to this could be that the HA statistic incorporates some details not taken into account by the RCI statistic (or by any other method based on distribution). While the RCI statistic is obtained by Jacobson et al. (1984, p. 14).


$$RCI=\frac{X_{i}-Y_{i}}{\sqrt{2{\left(S_{X}\sqrt{1-R_{XX}}\right)}^{2}}}$$

(*X_i_* = individual pre-test score; *Y_i_* = individual post-test score; *S_X_* = standard deviation of pre-test; *R_XX_* = reliability of test), the HA statistic (Jacobson et al., 1999, p.1173) is obtained by


$$HA=\frac{\left(Y_{i}-X_{i}\right)R_{DD}+\left(M_{Y}-M_{X}\right)\left(1-R_{DD}\right)}{\sqrt{2R_{DD}{\left(S_{X}\sqrt{1-R_{XX}}\right)}^{2}}}$$

(*M_X_* = mean of the pre-test scores; *M_Y_* = mean of the post-test scores; *R_DD_* = reliability of the pre-post differences).

The approach of Hageman and Arrindell (1999) tries to improve the accuracy of RCI by incorporating *the reliability of the pre-post differences*. Since working with pre-post differences has generated a lot of controversy among those who theorize about the psychometric properties of tests from classical test theory (due to the possible lack of reliability of this type of scores; see Lord, 1956, 1963; Rogosa and Willett, 1983), ignoring pre-post differences reliability does not seem the best way to proceed.

Therefore, the most remarkable difference between RCI and HA is that HA includes the reliability of differences (*R_DD_*). If *R_DD_* is perfect (*R_DD_* = 1), RCI and HA take identical values. If *R_DD_* is not perfect (*R_DD_* < 1), the HA formula does not clearly show what happens (because *R_DD_* plays a different part in the numerator and denominator), but both empirical and simulation studies indicate that as the value of *R_DD_* decreases, so does the value of HA, and that is why HA tends to make classifications more conservative than other distribution-based methods.

### How to estimate reliability

The confirmation that HA produces more conservative classifications than RCI (and more conservative than other distribution-based methods) is important considering that these methods tend to offer too high false positives rates.

But why do all distribution-based methods (including HA) offer excessively high false positives rates? The RCI and HA equations shown above (including the equations of other distribution-based methods) show that both statistics are based on the *standard error of measurement* (*SEM*), which is obtained by


$$SEM=S_{X}\sqrt{1-R_{XX}}$$

As we can see in the above equation, SEM depends on (a) the standard deviation of the pretest scores *S_X_* and (b) test reliability *R_XX_*. Now while there is only one way to calculate *S_X_*, there are many ways to calculate *R_XX_*. Each of these different approaches has advantages and disadvantages, but in the field of health sciences, the strategies most used are based on internal consistency (usually estimated by Cronbach’s coefficient alpha) (Cronbach, 1951) or on temporal stability (usually estimated by the test–retest correlation). Martinovich et al. (1996), after reflecting on the pros and cons of both strategies in the field of individual change assessment, recommended estimating reliability using internal consistency, especially for clinical populations, because test–retest reliability is reduced by the presence of true individual test–retest change, even without patients being on therapy during that period. and Wyrwich et al. (1999) also recommended estimating reliability by the alpha coefficient.

However, the psychometric literature contains numerous studies that advise against using alpha to estimate reliability (Schmitt, 1996; Bentler, 2009; Green and Yang, 2009; Revelle and Zinbarg, 2009; Sijtsma, 2009; Dunn et al., 2014; Crutzen and Peters, 2017). On the one hand, there is evidence that Cronbach’s alpha is not really an indicator of the internal consistency of a test (see, for example, Sijtsma, 2009). On the other hand, if a test is unidimensional, it is known that: (a) Only when the tau-equivalent assumption is assumed does the alpha coefficient produce results that are comparable to those of other measures of internal consistency (Graham, 2006), and (b) the reliability estimated through the alpha coefficient is higher than the one estimated using the test–retest correlation (Becker, 2000; Hogan et al., 2000; Green, 2003; Schmidt et al., 2003).

When this is considered, it seems that the recommendations given by Martinovich et al. (1996), and Wyrwich et al. (1999) would lead to evaluating statistically reliable change through the use of an underestimated value of SEM; and this is precisely what could justify, at least partially, the high false positives rate found in simulation studies. As a matter of fact, Pardo and Ferrer (2013) have proved that, when reliability is estimated through the test–retest correlation, both RCI and HA offer acceptable rates of false positives (which does not happen when reliability is estimated through Cronbach’s alpha).

Therefore, estimating reliability through the test–retest correlation implies not only working with a more realistic SEM, but also working with a value of SEM that has the direct consequence of reducing the false positive rate. But using the test–retest correlation to estimate the reliability of a test has a serious drawback: its value depends on the time-interval between first testing and the retest. If that interval is too short, there is a risk of overestimating the true reliability due to the recall of the subjects and their desire to be congruent; if the elapsed time is too long, there is a risk of underestimating true reliability because what is being measured may have changed. Since there is no way of knowing what the ideal time-interval should be between the two measurements, the estimates based on the test–retest correlation include an arbitrary component that is difficult to quantify and justify.

Accordingly, in this context, it is felt that the most reasonable measure for bypassing the interval issue would be to resort to alternative ways of estimating reliability. And among the available alternatives, McDonald’s omega (ω*_h_*) coefficient has been postulated as the most widely accepted and optimal measure of internal consistency (Shevlin et al., 2000; Zinbarg et al., 2005; Revelle and Zinbarg, 2009; Dunn et al., 2014). And what is more interesting, results obtained by Revelle and Zinbarg, (2009) in several groups of data show that ω*_h_* coefficient takes values systematically smaller than Cronbach’s alpha. Of course, this would indicate that ω*_h_* could be a good option for trying to reduce the rate of false positives associated with RCI and HA when reliability is estimated by Cronbach’s alpha.

### Objectives

This study has two main aims. First, we intend to make a detailed comparison of the RCI and HA statistics in various scenarios incorporating the use of a new way of estimating reliability (ω*_h_*). This will allow us to assess the false positive and false negative rates associated with each method in many new scenarios.

Second, since neither RCI nor HA can be calculated with the most widely used computer programs, we put forward to offer to non-expert users can Excel macro to easily calculate these statistics given the conceptual advantage of the HA method, it does not seem reasonable to suggest that the choice for RCI above HA should be based solely on the fact that it is easier to calculate RCI, as suggested by Ronk et al. (2016).

## Methods

To evaluate the false positive and false negative rates of RCI and HA methods, a pre-post design were simulated. In this design, a first measurement is obtained before treatment (*X,* or pre-treatment score) and a second measurement is obtained after treatment (*Y,* or post-treatment score), in the same group of subjects.

The simulated scores were generated assuming no change (null effect size) and different changes (different effect sizes) between pre- and post-measures. The general simulated scenario was a 10 items pre-test measurement (pre-test score was computed by the arithmetic mean of these 10 items), with equal factorial loadings (a *tau*-equivalent scenario in classic test theory), to estimate the reliability (by internal consistency). A post-test score fixed to Pearson’s correlation coefficient of 0.8 (*R_XY_* = 0.80) with the pre-test score to represent common levels of test–retest reliability (Cicchetti, 1994) (for a detailed comparison of the effects of different test–retest correlation sizes, see Pardo and Ferrer, 2013; Ferrer and Pardo, 2019). To generate the different simulated situations, we used four criteria:

- *The shape of the pre- and post-treatment score distribution*. Given that moderate and severe deviations from normality are often found in applied contexts (Micceri, 1989; Blanca et al., 2013), we simulated different values for skewness, ranging from extremely negative to extremely positive, and kurtosis. Using the Pearson distribution system as a reference, we generated five different distributions, four of which represent different degrees of deviation from normality. The degree of deviation from normality was controlled manipulating the value of the skewness (*g*_1_) and kurtosis (*g*_2_) indexes in the following manner: (*a*) *normal distribution: g*_1_ = 0, *g*_2_ = 0; (*b*) *negative very asymmetric distribution: g*_1_ = −4, *g*_2_ = 18; (*c*) *negative moderately asymmetric distribution: g*_1_ = −2, *g*_2_ = 4; (*d*) *positive moderately asymmetric distribution: g*_1_ = 2, *g*_2_ = 4; (*e*) *positive very asymmetric distribution: g*_1_ = 4, *g*_2_ = 18.
- *The sample size* (*n*): 25, 50, 100. We selected different sample sizes with the intention of representing what is known in the clinical field as small, medium, and large sizes (see, for example, Crawford and Howell, 1998).
- *The effect size* (δ): 0, 0.2, 0.5, 0.8, 1.1, 1.4, 1.7 and 2 standard deviations of the differences. These values correspond to the systematic increase in the post-scores *Y* expressed in standard deviation of pre-post differences, in the different simulated conditions. Because individual changes have greater variability than average changes, we have chosen effect sizes that range from small (0.2 standard deviations) to very large (2 standard deviations), with increases of 0.3 points. First effect size (0.0) represent a non-change scenario to estimate the false positive rates; the remaining values correspond to the systematic increase on the post scores *Y* in the different simulated conditions to estimate the false negative rates.
  For a pre-post design, the effect size is usually computed as the standardized pre-post difference (Cohen, 1988). However, standardization can be carried out in two different ways: by dividing the mean of the pre-post differences between the standard deviation of pre-test scores (*S_X_*), or between the standard deviation of pre-post differences (*S_D_*). Following recommendations of some authors (Cohen, 1988; Cumming and Finch, 2001), we use the standard deviation of the pre-test (*S_X_*) as a standardizer since the natural reference for thinking about original scores is the variability in the pre-test scores (*S_X_*).
- *Factorial loadings in the pre-test* (*λ*): 0.40, 0.50 and 0.60. These values were selected to represent common values observed in psychometrics factorial analyses (Peterson, 2000) and were used to estimate reliability (by internal consistency) using Cronbach’s alpha and McDonald’s omega coefficients.

A total of 5(distributions) × 3(sample sizes) × 8(effect sizes) × 3(factorial loadings) = 360 conditions were defined combining these four criteria, and a thousand samples were generated for each of these 120 conditions. Details of the simulation are included in the additional documentation (see supplementary files).

For data analysis, we made the necessary computations to obtain RCI and HA in each simulated sample. Finally, the performance of each statistic was assessed by applying the corresponding criterion, that is, recording the observed false positive and false negative rates. We considered that a false positive occurred when, with effect size = 0, a pre-post difference exceeded the corresponding cut-off point established as the change criterion: ≥ 1.65, in absolute value, for both RCI and HA, so make false positives and negatives rates were also comparable. We considered that a false negative occurred when, with effect size >0, a pre-post difference did not exceed the corresponding cut-off point. The 1.65 criterion corresponds to the reference point in a normal distribution that should be below the distribution in 95% of the cases, that is, the cut-off point at which one would expect to observe a false-positive rate of approximately 5%.For simulation, and for many of the calculations, we used the MATLAB 20009b program. To compute the mean results from the samples of each condition, we used the IBM SPSS Statistics v. 22 program.

## Results

Since publishing limitations prevent us from including all the results generated by the collection of simulated conditions, the present report only includes percentages of false negatives and false positives.

Table 1 offers the percentage of false positives (when effect size = 0) and false negatives (when effect size >0) associated with the RCI statistic. Table 2 offers the same percentages for the HA statistic. These percentages were obtained by calculating the number of false positives and false negatives in the 1,000 samples for each condition. Following the liberal criterion of Bradley (1978), percentages of false positives between 2.5 and 7.5% were considered acceptable (and shaded). Following a similar logic, the percentages of false negatives under 25% were considered correct (and shaded).

**Table 1:** RCI: mean (standard deviation) percentage of false positives and false negatives.

			Effect size (*δ*)							
	*λ*	*g*_1_, *g*_2_	0	0.2	0.5	0.8	1.1	1.4	1.7	2.0
*n* = 25	0.4	0, 0	13.9 (0.08)	84.7 (0.08)	78.5 (0.10)	68.1 (0.13)	55.3 (0.15)	41.5 (0.15)	29.5 (0.15)	18.8 (0.13)
		2, 4	13.1 (0.07)	86.4 (0.07)	82.9 (0.08)	74.9 (0.11)	63.0 (0.18)	46.3 (0.23)	31.1 (0.23)	18.9 (0.20)
		−2, 4	13.5 (0.07)	85.1 (0.08)	77.7 (0.14)	61.1 (0.21)	44.4 (0.24)	31.7 (0.22)	22.8 (0.18)	17.0 (0.15)
		4, 18	11.4 (0.06)	88.8 (0.06)	85.8 (0.11)	71.4 (0.26)	54.9 (0.32)	40.3 (0.33)	29.0 (0.31)	20.0 (0.27)
		−4, 18	11.0 (0.06)	87.3 (0.08)	77.2 (0.19)	58.7 (0.32)	42.8 (0.35)	31.2 (0.33)	22.5 (0.28)	16.5 (0.24)
	0.5	0, 0	20.6 (0.09)	77.6 (0.10)	68.9 (0.11)	55.5 (0.14)	40.0 (0.14)	26.3 (0.14)	16.1 (0.12)	8.7 (0.09)
		2, 4	17.7 (0.08)	82.0 (0.08)	76.8 (0.10)	64.2 (0.17)	45.2 (0.22)	28.0 (0.21)	15.6 (0.16)	7.8 (0.12)
		−2, 4	18.8 (0.09)	78.8 (0.10)	65.7 (0.18)	45.6 (0.22)	30.3 (0.19)	20.7 (0.15)	14.4 (0.12)	10.4 (0.10)
		4, 18	13.2 (0.07)	86.9 (0.07)	77.9 (0.20)	58.1 (0.32)	41.5 (0.33)	29.2 (0.31)	21.1 (0.28)	14.7 (0.24)
		−4, 18	13.2 (0.07)	84.1 (0.09)	65.9 (0.26)	44.0 (0.34)	30.0 (0.33)	21.5 (0.29)	15.4 (0.24)	11.1 (0.19)
	0.6	0, 0	30.3 (0.11)	67.4 (0.11)	57.5 (0.12)	42.3 (0.13)	27.2 (0.12)	15.4 (0.10)	7.7 (0.07)	3.5 (0.04)
		2, 4	23.5 (0.09)	76.1 (0.09)	67.9 (0.14)	47.7 (0.20)	28.6 (0.19)	15.7 (0.15)	7.9 (0.11)	3.3 (0.07)
		−2, 4	24.1 (0.09)	72.3 (0.11)	53.7 (0.20)	33.0 (0.20)	21.6 (0.16)	14.5 (0.12)	10.0 (0.10)	7.0 (0.08)
		4, 18	16.2 (0.08)	83.6 (0.08)	64.7 (0.28)	42.7 (0.33)	29.4 (0.31)	20.4 (0.27)	13.9 (0.23)	9.4 (0.20)
		−4, 18	15.7 (0.07)	80.4 (0.11)	54.1 (0.31)	34.4 (0.33)	23.3 (0.30)	16.5 (0.26)	12.0 (0.21)	8.8 (0.18)
*n* = 50	0.4	0, 0	14.2 (0.05)	84.5 (0.06)	78.5 (0.07)	67.8 (0.09)	54.4 (0.10)	40.4 (0.11)	27.7 (0.10)	17.3 (0.09)
		2, 4	12.3 (0.05)	86.9 (0.05)	83.5 (0.05)	77.4 (0.07)	66.0 (0.13)	48.0 (0.18)	29.0 (0.18)	15.6 (0.14)
		−2, 4	12.3 (0.05)	86.8 (0.05)	80.4 (0.09)	64.5 (0.16)	44.3 (0.19)	29.7 (0.15)	20.6 (0.11)	14.9 (0.08)
		4, 18	10.1 (0.04)	89.9 (0.04)	88.4 (0.05)	79.6 (0.17)	60.5 (0.28)	40.9 (0.30)	26.4 (0.27)	15.9 (0.21)
		−4, 18	10.3 (0.05)	88.9 (0.05)	82.7 (0.12)	64.6 (0.27)	44.0 (0.32)	29.1 (0.29)	19.3 (0.23)	12.5 (0.17)
	0.5	0, 0	21.1 (0.06)	77.1 (0.07)	68.7 (0.08)	55.2 (0.09)	40.1 (0.10)	26.2 (0.09)	15.3 (0.08)	7.8 (0.06)
		2, 4	17.5 (0.06)	82.1 (0.06)	77.7 (0.07)	66.2 (0.12)	45.2 (0.17)	25.4 (0.16)	12.5 (0.11)	8.8 (0.07)
		−2, 4	17.5 (0.06)	80.4 (0.07)	68.1 (0.13)	48.7 (0.17)	28.5 (0.14)	18.8 (0.10)	13.4 (0.07)	9.4 (0.06)
		4, 18	12.6 (0.05)	87.4 (0.05)	82.7 (0.11)	61.5 (0.28)	39.6 (0.31)	24.9 (8.27)	15.3 (0.22)	9.2 (0.17)
		−4, 18	12.4 (0.05)	85.9 (0.06)	72.3 (0.19)	46.1 (0.31)	27.8 (0.28)	17.4 (0.22)	11.4 (0.17)	7.8 (0.13)
	0.6	0, 0	29.9 (0.07)	67.9 (0.07)	57.6 (0.09)	42.3 (0.09)	27.0 (0.09)	15.0 (0.7)	7.1 (0.05)	3.0 (0.03)
		2, 4	23.3 (0.07)	76.7 (0.07)	69.1 (0.09)	47.0 (0.16)	25.3 (0.14)	12.3 (0.09)	8.5 (0.04)	2.1 (0.02)
		−2, 4	23.3 (0.07)	73.0 (0.08)	53.0 (0.15)	30.0 (0.14)	18.8 (0.10)	12.4 (0.07)	8.4 (0.06)	5.7 (0.05)
		4, 18	14.8 (0.05)	84.9 (0.05)	71.0 (0.21)	41.7 (0.30)	23.5 (0.26)	13.8 (0.19)	08.1 (0.14)	4.8 (0.10)
		−4, 18	14.8 (0.05)	82.0 (0.07)	57.5 (0.26)	30.6 (0.29)	17.5 (0.22)	10.7 (0.17)	7.2 (0.13)	5.2 (0.10)
*n* = 100	0.4	0, 0	14.0 (0.4)	84.9 (0.04)	78.8 (0.05)	68.2 (0.06)	54.8 (0.07)	40.7 (0.08)	27.7 (0.07)	17.1 (0.06)
		2, 4	12.1 (0.03)	87.1 (0.03)	83.9 (0.04)	78.0 (0.05)	66.8 (0.09)	46.9 (0.14)	25.7 (0.13)	11.9 (0.08)
		−2, 4	12.1 (0.03)	87.2 (0.04)	81.5 (0.06)	65.9 (0.12)	43.8 (0.14)	28.2 (0.10)	19.6 (0.07)	14.5 (0.05)
		4, 18	9.2 (0.03)	90.8 (0.03)	89.6 (0.03)	84.8 (0.10)	66.7 (0.23)	42.2 (0.27)	23.6 (0.23)	12.3 (0.15)
		−4, 18	9.3 (0.03)	90.1 (0.03)	85.5 (0.07)	69.0 (0.20)	42.4 (0.27)	22.7 (0.22)	12.4 (0.14)	8.3 (0.14)
	0.5	0, 0	21.1 (0.05)	77.1 (0.05)	68.4 (0.06)	54.8 (0.07)	39.3 (0.07)	25.0 (0.06)	14.0 (0.05)	6.9 (0.03)
		2, 4	17.4 (0.04)	82.2 (0.04)	77.8 (0.05)	66.4 (0.09)	43.0 (0.14)	21.6 (0.11)	9.9 (0.06)	4.2 (0.03)
		−2, 4	17.2 (0.04)	80.8 (0.05)	69.3 (0.09)	45.5 (0.13)	27.2 (0.10)	18.2 (0.06)	12.9 (0.05)	9.1 (0.04)
		4, 18	12.0 (0.03)	88.0 (0.03)	85.5 (0.06)	65.4 (0.23)	37.3 (0.26)	19.0 (0.20)	8.5 (0.12)	4.8 (0.06)
		−4, 18	11.8 (0.03)	86.8 (0.04)	76.3 (0.12)	46.7 (0.26)	22.5 (0.22)	12.0 (0.14)	7.6 (0.08)	5.6 (0.05)
	0.6	0, 0	29.9 (0.05)	67.9 (0.5)	57.6 (0.06)	42.2 (0.06)	26.6 (0.06)	14.3 (0.05)	6.6 (0.03)	2.7 (0.02)
		2, 4	23.1 (0.04)	76.6 (0.04)	69.5 (0.06)	46.4 (0.12)	22.2 (0.09)	10.2 (0.04)	4.5 (0.02)	1.9 (0.01)
		−2, 4	23.0 (0.04)	73.6 (0.05)	54.4 (0.11)	29.0 (0.09)	17.7 (0.06)	11.9 (0.04)	8.0 (0.03)	5.3 (0.03)
		4, 18	14.1 (0.04)	85.6 (0.04)	76.3 (0.15)	41.8 (0.27)	19.6 (0.20)	9.9 (0.13)	5.4 (0.08)	3.1 (0.04)
		−4, 18	14.3 (0.04)	83.3 (0.05)	62.0 (0.19)	26.5 (0.23)	12.2 (0.14)	7.3 (0.09)	5.1 (0.05)	3.9 (0.03)

**Table 2:** HA: mean (standard deviation) percentage of false positives and false negatives.

			Effect size (*δ*)							
	*λ*	*g*_1_, *g*_2_	0	0.2	0.5	0.8	1.1	1.4	1.7	2.0
*n* = 25	0.4	0, 0	5.3 (0.15)	89.5 (0.21)	69.9 (0.34)	50.6 (0.39)	39.1 (0.43)	34.2 (0.45)	32.3 (0.45)	31.6 (0.46)
		2, 4	6.7 (0.14)	89.3 (0.18)	75.5 (0.29)	55.0 (0.40)	40.4 (0.44)	36.0 (0.46)	34.8 (0.47)	34.6 (0.47)
		−2, 4	5.9 (0.10)	91.0 (0.18)	71.5 (0.32)	48.5 (0.39)	39.8 (0.42)	36.3 (0.44)	34.4 (0.45)	33.3 (0.46)
		4, 18	7.5 (0.10)	90.9 (0.15)	74.7 (0.27)	51.9 (0.40)	40.6 (0.45)	38.1 (0.47)	37.1 (0.47)	36.8 (0.48)
		−4, 18	7.5 (0.11)	90.2 (0.16)	72.8 (0.32)	47.8 (0.42)	41.2 (0.45)	40.0 (0.45)	40.0 (0.46)	38.5 (0.46)
	0.5	0, 0	8.9 (0.13)	85.1 (0.18)	60.8 (0.289)	36.6 (0.30)	21.6 (0.30)	14.7 (0.30)	11.8 (0.30)	10.8 (0.30)
		2, 4	11.1 (0.14)	85.6 (0.17)	68.9 (0.28)	39.8 (0.33)	21.8 (0.33)	16.2 (0.34)	14.6 (0.34)	14.3 (0.34)
		−2, 4	11.2 (0.13)	84.2 (0.18)	59.5 (0.30)	33.9 (0.31)	24.0 (0.31)	19.3 (0.32)	16.9 (0.33)	15.4 (0.33)
		4, 18	10.1 (0.13)	88.5 (0.16)	73.6 (0.30)	40.3 (0.38)	30.3 (0.41)	27.7 (0.42)	26.4 (0.43)	26.0 (0.43)
		−4, 18	9.9 (0.11)	86.6 (0.17)	61.6 (0.34)	36.0 (0.39)	29.5 (0.41)	28.1 (0.41)	27.3 (0.42)	26.8 (0.42)
	0.6	0, 0	17.1 (0.11)	77.8 (0.14)	54.5 (0.19)	29.5 (0.18)	13.4 (0.14)	5.4 (0.11)	2.4 (0.09)	1.3 (0.09)
		2, 4	16.2 (0.12)	81.6 (0.14)	64.9 (0.22)	30.5 (0.24)	13.8 (0.22)	8.0 (0.22)	5.9 (0.22)	5.5 (0.22)
		−2, 4	16.3 (0.12)	78.0 (0.17)	50.1 (0.27)	25.1 (0.23)	16.0 (0.21)	11.2 (0.21)	8.4 (0.21)	6.7 (0.21)
		4, 18	12.9 (0.10)	86.2 (0.12)	62.5 (0.31)	28.3 (0.32)	19.9 (0.33)	17.1 (0.34)	15.5 (0.35)	14.7 (0.35)
		−4, 18	12.5 (0.11)	82.7 (0.16)	48.0 (0.36)	25.2 (0.34)	18.8 (0.33)	17.4 (0.33)	16.6 (0.34)	16.0 (0.34)
*n* = 50	0.4	0, 0	3.8 (0.14)	90.6 (0.21)	63.4 (0.34)	37.2 (0.36)	24.9 (0.37)	20.7 (0.38)	19.5 (0.39)	19.2 (0.39)
		2, 4	4.5 (0.09)	90.8 (0.15)	72.1 (0.30)	44.7 (0.39)	30.7 (0.42)	27.8 (0.44)	27.2 (0.44)	27.1 (0.44)
		−2, 4	4.3 (0.10)	93.1 (0.17)	67.3 (0.34)	40.5 (0.38)	32.2 (0.41)	29.1 (0.42)	27.5 (0.43)	26.7 (0.43)
		4, 18	5.0 (0.07)	92.5 (0.14)	81.5 (0.28)	50.8 (0.40)	38.7 (0.45)	36.4 (0.47)	35.6 (0.47)	35.4 (0.47)
		−4, 18	6.0 (0.10)	92.6 (0.13)	72.8 (0.31)	58.0 (0.41)	35.2 (0.43)	33.8 (0.44)	33.0. (44)	32.4 (0.45)
	0.5	0, 0	6.5 (0.07)	88.2 (0.12)	62.0 (0.21)	31.5 (0.20)	12.5 (0.16)	4.9 (0.13)	2.5 (0.13)	2.0 (0.13)
		2, 4	8.3 (0.09)	88.3 (0.12)	71.5 (0.22)	35.6 (0.27)	14.2 (0.25)	9.0 (0.25)	7.6 (0.25)	7.4 (0.26)
		−2, 4	8.8 (0.09)	88.3 (0.13)	61.5 (0.26)	29.3 (0.25)	18.2 (0.25)	13.2 (0.26)	10.6 (0.26)	9.2 (0.26)
		4, 18	8.1 (0.07)	89.8 (0.12)	76.3 (0.26)	32.6 (0.33)	20.5 (0.35)	18.0 (0.36)	17.0 (0.36)	16.5 (0.36)
		−4, 18	8.4 (0.10)	89.1 (0.14)	63.0 (0.31)	27.7 (0.34)	21.1 (0.34)	19.5 (0.35)	18.5 (0.35)	17.7 (0.36)
	0.6	0, 0	16.0 (0.08)	79.7 (0.09)	57.9 (0.13)	31.1 (0.13)	12.7 (0.09)	4.0 (0.05)	1.2 (0.03)	0.4 (0.03)
		2, 4	14.4 (0.08)	83.7 (0.08)	68.2 (0.14)	28.6 (0.17)	9.7 (0.12)	4.1 (0.12)	2.1 (0.12)	1.7 (0.12)
		−2, 4	14.5 (0.07)	80.9 (0.11)	50.1 (0.20)	21.9 (0.14)	12.0 (0.12)	7.3 (0.11)	4.4 (0.11)	2.9 (0.11)
		4, 18	11.0 (0.07)	87.3 (0.11)	67.8 (0.26)	20.7 (0.23)	10.8 (0.22)	8.5 (0.23)	7.2 (0.23)	6.5 (0.23)
		−4, 18	11.2 (0.08)	85.8 (0.12)	52.3 (0.31)	20.0 (0.27)	13.7 (0.26)	12.0 (0.26)	11.0 (0.27)	10.3 (0.27)
*n* = 100	0.4	0, 0	2.0 (0.10)	91.7 (0.20)	56.3 (0.32)	24.8 (0.30)	13.3 (0.29)	10.7 (0.30)	10.2 (0.30)	10.2 (0.30)
		2, 4	3.4 (0.09)	91.7 (0.15)	67.5 (0.31)	32.2 (0.36)	18.8 (0.36)	16.9 (0.37)	16.6 (0.37)	16.5 (0.37)
		−2, 4	3.0 (0.08)	92.8 (0.21)	64.8 (0.33)	31.9 (0.35)	23.6 (0.37)	20.7 (0.38)	19.6 (0.38)	19.2 (0.38)
		4, 18	3.8 (0.08)	92.9 (0.15)	77.2 (0.32)	40.3 (0.40)	30.1 (0.43)	28.7 (0.44)	28.3 (0.44)	28.2 (0.44)
		-4, 18	3.8 (0.06)	92.7 (0.16)	71.3 (0.33)	35.9 (0.40)	30.6 (0.42)	29.1 (0.43)	28.4 (0.43)	27.9 (0.43)
	0.5	0, 0	5.3 (0.04)	90.3 (0.06)	63.7 (0.15)	29.9 (0.15)	9.8 (0.08)	2.5 (0.04)	0.6 (0.03)	0.2 (0.03)
		2, 4	7.5 (0.07)	89.5 (0.07)	72.5 (0.17)	30.5 (0.20)	7.9 (0.13)	3.1 (0.13)	2.0 (0.13)	1.9 (0.13)
		−2, 4	7.2 (0.06)	90.5 (0.11)	61.6 (0.21)	22.6 (0.14)	11.0 (0.12)	6.0 (0.11)	3.4 (0.11)	2.1 (0.11)
		4, 18	7.2 (0.06)	91.0 (0.11)	80.5 (0.21)	27.0 (0.25)	12.6 (0.26)	10.2 (0.27)	9.1 (0.27)	8.7 (0.27)
		−4, 18	7.0 (0.6)	90.2 (0.13)	64.7 (0.28)	20.8 (0.27)	14.7 (0.28)	12.9 (0.28)	11.9 (0.28)	11.1 (0.29)
	0.6	0, 0	15.1 (0.05)	80.4 (0.06)	59.2 (0.08)	31.9 (0.09)	12.5 (0.06)	3.7 (0.03)	0.9 (0.01)	0.2 (0.00)
		2, 4	13.9 (0.05)	84.1 (0.05)	70.4 (0.08)	27.8 (0.11)	8.1 (0.04)	2.4 (0.01)	0.5 (0.00)	0.1 (0.00)
		−2, 4	13.9 (0.05)	83.0 (0.06)	54.2 (0.14)	21.4 (0.07)	11.1 (0.05)	5.8 (0.03)	2.9 (0.02)	1.4 (0.01)
		4, 18	10.1 (0.04)	89.0 (0.06)	74.9 (0.19)	18.4 (0.17)	7.9 (0.16)	5.5 (0.16)	4.2 (0.17)	3.5 (0.17)
		−4, 18	10.3 (0.04)	87.7 (0.07)	56.3 (0.24)	12.5 (0.14)	7.1 (0.12)	5.4 (0.12)	4.3 (0.12)	3.5 (0.12)

Information regarding the accuracy of the performed simulation, provided evidence that the simulated data reproduced the imposed conditions reasonably well (see Simulation Tables in the supplementary files). However, as in other studies (Pardo and Ferrer, 2013; Ferrer and Pardo, 2019), only skewness and kurtosis deviated from what was expected (the smaller the sample size, the greater the deviation). This occurred because the standard errors of the statistics used to evaluate skewness and kurtosis increased as the sample size decreased (Wright and Herrington, 2011).

### False positives

Percentages of false positives obtained with the RCI statistic were systematically higher than the standard nominal level: where one would have expected to find values around 5%, we found values that ranged from 9.2 to 30.3%. These percentages were not significantly altered, neither by the shape of the simulated distributions nor by the different sample sizes used in the present study.

The percentages of false positives obtained with the HA statistic were more acceptable; in fact, these percentages took correct values when *λ* = 0.4 (regardless of the shape of the distribution and of the sample size) and when *λ* = 0.5 if *n* = 100 (regardless of the shape of the distribution). In the rest of the simulated conditions, percentages higher than the nominal level were obtained, although in no case were values observed as high as those obtained with the RCI statistic.

### False negatives

RCI and HA were better comparable in terms of the percentage of false negatives they generated. With the RCI statistic, these percentages tended to improve as the value of λ increased; but correct percentages were only obtained if δ was greater than 1. With the HA statistic, the percentages of false negatives were also better when *λ* equaled 0.5 or 0.6 than when it equaled 0.4, but some correct percentages were also obtained when *δ* = 0.8. It also occurred that the percentages of false negatives improved slightly as sample size increased (this occurred in relation to both the RCI and the HA statistic).

## Discussion

The aim of the present study was to estimate the rate of false positives and false negatives associated with RCI and HA, incorporating the use of a new way of estimating reliability. Since false positives and false negatives represent classification errors, it would be reasonable to expect a good diagnostic method to be able to make proper classifications while maintaining low rates of false positives and false negatives.
It is commonly assumed that the false positive rate should be around 0.05. How low the false negative rate should be is also a subjective issue, but in applied research and clinical practice, it is common to consider that this rate should not exceed 20% (Cohen, 1988, 1992). Taking these two conventional values as a reference (5 and 20%, respectively), the results of the present study indicate that:

- RCI offers unacceptable false positives rates in all simulated conditions. As this occurs when reliability is estimated by Cronbach’s alpha coefficient (Ferrer and Pardo, 2019), when reliability is estimated by the *omega_h_* coefficient, false positive rates associated with RCI take values well above the nominal value. These unacceptable values increase slightly when λ increases. When the samples come from normal distributions, they also tend to be higher than when they come from asymmetric distributions.
- HA offers acceptable false positive rates in some simulated conditions. When *λ* = 0.4, all false positive rates take correct values (regardless of the sample size and the shape of the simulated distributions). When the value of *λ* increases, the false positives rate also increases. The presence of acceptable rates of false positives in several of the simulated conditions indicates that significantly better results are obtained when using the *omega_h_* coefficient to estimate reliability than when estimating reliability with the alpha coefficient. It is true that estimating reliability with the test–retest correlation provides better results than estimations through alpha (Ferrer and Pardo, 2019); however, estimates based on the *omega_h_* coefficient do not have the aforementioned drawbacks that estimates based on the test–retest correlation have.
- Both RCI and HA offer unacceptable rates of false negative. All false negative rates decrease as the effect size increases: this is to be expected if we take into account that the greater the mean of the pre-post differences, the greater a randomly selected individual difference is to be expected. But, even though false negative rates should not exceed 20% (25% applying a criterion similar to the criterion of Bradley for false positives), with RCI statistic rates were found that ranged from 67.4 to 90.8% when the effect size was 0.2 (a small effect size according to Cohen’s criteria); and rates that ranged from 12.2 to 66.8% when the effect size was 0.8 (a large effect size according to Cohen’s criteria). With the HA statistic, rates were found that ranged from 77.8 to 93.1% when the effect size was 0.2; and rates that ranged from 12.5 to 58.0% when the effect size was 0.8. Therefore, neither RCI nor HA perform well regarding false negative rates.

Nevertheless, to be able to correctly interpret these results, it is necessary to take into account some considerations related to Cohen’s standardized difference (δ) and the reference values specifically proposed by Cohen (1988) to interpret *δ*. The cut-off points proposed by Cohen to identify small, medium, and large effect sizes (0.2, 0.5, and 0.8, respectively) do not seem to have been sufficiently justified in order to be accepted as reference values. Indeed, both Cohen and other experts recommended using these cut-off points as mere guides and not as fixed, rigid criteria (Cohen, 1992; Snyder and Lawson, 1993; Thompson, 2002). Ferguson (2009), for example, based on previous reviews (Franzblau, 1958; Lipsey and Hurley, 2009), proposed reference values that depart markedly from those proposed by Cohen. Ferguson’s specific proposal is as follows: 0.41 for a *minimum* effect, 1.15 for a *moderate* effect and 2.70 for a *strong* effect. It is clear that the criteria initially proposed by Cohen (criteria considered valid by most researchers) differ meaningfully from those proposed by Ferguson.

One illustration will suffice. At the evaluation level, for example, the observation of a large therapeutic effect (*δ* = 0.80 according to Cohen) in the positive direction (i.e., less complaints/negative affect or greater well-being/positive affect) suggests that 19.9% of the clients obtain pre-post differences that represent a reliable change (i.e., differences that surpass the cutoff point 1.645, the 95th percentile of a normal distribution). When a large effect size is achieved following the directives of Ferguson (*δ* = 2.70), 85.4% of clients obtain pre-post differences that represent a reliable change (in calculating these percentages we assume that pre-post differences are normally distributed).

These considerations about the cut-off points used to define small, medium and large effect sizes lead to the following conclusion: taking 1.15 (instead of 0.5) as a reference value for an effect of medium size, the false positive rates associated with the HA statistic seem quite correct when *λ* > 0.4. Therefore, the false negative rate obtained does not seem as unacceptable as it initially appeared.

Finally, classifications resulting from the application of these cutoffs could be improved if the results obtained by applying distribution-based methods such as RCI and HA were supplemented by information provided by anchor-based methods (Barrett et al., 2008; de Vet and Terwee, 2010; Houweling, 2010; Turner et al., 2010) or the cumulative proportion of responders (Farrar et al., 2006; McLeod et al., 2011; Wyrwich et al., 2013). This, however, is an area in need of further research.

## Conclusion

The objective of the present study was to learn about the false negative and false negative rates associated with two distribution-based methods (RCI and HA) designed to evaluate individual change (reliable change) in pre-post designs. The novelty of this study is that reliability has been estimated by the *omega_h_* coefficient rather than with the alpha coefficient or the test–retest correlation.

Regarding the rate of false positives, only the HA statistic provides acceptable results. Regarding the rate of false negatives, both statistics offer similar results, and both can claim to offer acceptable rates when Ferguson’s stringent criteria are used to define effect sizes rather than when the conventional criteria advanced by Cohen is employed.

Since the HA statistic seems to be a better option than the RCI statistic, we have developed an Excel macro (see Supplementary files) so that the greater complexity of calculating HA does not represent an obstacle for the non-expert user.

The methods used to establish the minimally reliable change analyzed in the present study offer an opportunity to assess the change experienced by a person or a group of people as a consequence of an intervention. So far, we have used the clinical context as an example, but this approach could be used in a wide range of contexts, e.g., in educational, community, and/or social intervention areas, to assess the effectiveness of skills training program, to test interventions in the organizational area, to evaluate cognitive stimulation and/or learning programs, etc.

However, some considerations must be taken into account before applying these reliable change measures. This approach is used in pre-post research designs; the trait or symptoms of interest should be susceptible to change as a result of the intervention; the scales used must have evidence of validity and sufficient reliability (because reliability is an important parameter within the equation for its estimation); and certain minimum reference information must be available or there must be a sufficient sample to estimate this information. For example, it could be applied with scales commonly used in psychotherapeutic contexts, e.g., the Beck Depression Inventory (BDI), the Hamilton Anxiety Rating Scale (HAM-A), and the Global Assessment of Functioning (GAF).

## Data availability statement

The datasets presented in this study can be found in online repositories. The names of the repository/repositories and accession number(s) can be found in the article/supplementary material.

## Author contributions

RF-U: conceptualization, data curation, formal analysis, methodology, writing – original draft, writing – review, and editing. AP: conceptualization, formal analysis, methodology, supervision, validation, writing – original draft, writing – review, and editing. WA: conceptualization, supervision, validation, writing – review, and editing. GP-G: formal analysis, writing – review, editing, and validation. All authors contributed to the article and approved the submitted version.

## Conflict of interest

The authors declare that the research was conducted in the absence of any commercial or financial relationships that could be construed as a potential conflict of interest.

## Publisher’s note

All claims expressed in this article are solely those of the authors and do not necessarily represent those of their affiliated organizations, or those of the publisher, the editors and the reviewers. Any product that may be evaluated in this article, or claim that may be made by its manufacturer, is not guaranteed or endorsed by the publisher.

## Abbreviations

RCI: The reliable change index
EN: The Edwards-Nunnally method
GLN: The Gulliksen-Lord-Novick method
HLM: The hierarchical linear modeling
HA: The Hageman-Arrindell method
SEM: Standard error of measurement
